# *miR-190a-5p* Partially Represses the Abnormal Electrical Activity of SCN3B in Cardiac Arrhythmias by Downregulation of IL-2

**DOI:** 10.3389/fcvm.2021.795675

**Published:** 2022-01-10

**Authors:** Qianqian Li, Ziguan Zhang, Shanshan Chen, Zhengrong Huang, Mengru Wang, Mengchen Zhou, Chenguang Yu, Xiangyi Wang, Yilin Chen, Dan Jiang, Dunfeng Du, Yufeng Huang, Xin Tu, Zhishui Chen, Yuanyuan Zhao

**Affiliations:** ^1^Department of Obstetrics and Gynecology, Genetics and Prenatal Diagnosis Center, The First Affiliated Hospital of Zhengzhou University, Zhengzhou, China; ^2^Key Laboratory of Molecular Biophysics of the Ministry of Education, Cardio-X Center, College of Life Science and Technology and Center for Human Genome Research, Huazhong University of Science and Technology, Wuhan, China; ^3^Department of Cardiology, Xiamen Key Laboratory of Cardiac Electrophysiology, Xiamen Institute of Cardiovascular Diseases, The First Affiliated Hospital of Xiamen University, School of Medicine, Xiamen University, Xiamen, China; ^4^Key Laboratory for Molecular Diagnosis of Hubei Province, Tongji Medical College, The Central Hospital of Wuhan, Huazhong University of Science and Technology, Wuhan, China; ^5^Department of Cardiology, Tongji Medical College, Union Hospital, Huazhong University of Science and Technology, Wuhan, China; ^6^Institute of Organ Transplantation, Tongji Medical College, Tongji Hospital, Huazhong University of Science and Technology, Wuhan, China; ^7^Key Laboratory of Organ Transplantation, Ministry of Education, Chinese Academy of Medical Sciences, Wuhan, China; ^8^NHC Key Laboratory of Organ Transplantation, Chinese Academy of Medical Sciences, Wuhan, China; ^9^Key Laboratory of Organ Transplantation, Chinese Academy of Medical Sciences, Wuhan, China; ^10^Precision Medical Center, Wuhan Children's Hospital (Wuhan Maternal and Child Healthcare Hospital), Tongji Medical College, Huazhong University of Science and Technology, Wuhan, China

**Keywords:** cardiac arrhythmias, inflammation, interleukin-2, microRNA, sodium channel current, SCN3B

## Abstract

Cardiac arrhythmias (CAs) are generally caused by disruption of the cardiac conduction system; interleukin-2 (IL-2) is a key player in the pathological process of CAs. This study aimed to investigate the molecular mechanism underlying the regulation of IL-2 and the sodium channel current of sodium voltage-gated channel beta subunit 3 (SCN3B) by *miR-190a-5p* in the progression of CAs. ELISA results suggested the concentration of peripheral blood serum IL-2 in patients with atrial fibrillation (AF) to be increased compared to that in normal controls; fluorescence *in situ* hybridization indicated that the expression of IL-2 in the cardiac tissues of patients with AF to be upregulated and that *miR-190a-5p* to be downregulated. Luciferase reporter assay, quantitative real-time-PCR, and whole-cell patch-clamp experiments confirmed the downregulation of IL-2 by *miR-190a-5p* and influence of the latter on the sodium current of SCN3B. Overall, *miR-190a-5p* suppressed the increase in SCN3B sodium current caused by endogenous IL-2, whereas *miR-190a-5p* inhibitor significantly reversed this effect. IL-2 was demonstrated to be directly regulated by *miR-190a-5p*. We, therefore, concluded that the *miR-190a-5p*/IL-2/SCN3B pathway could be involved in the pathogenesis of CAs and *miR-190a-5p* might acts as a potential protective factor in pathogenesis of CAs.

## Introduction

Interleukin-2 (IL-2), a proinflammatory factor, is predominantly secreted by activated T lymphocytes (1). The vital role of IL-2 is to stimulate the proliferation of T cells and generate effector and memory T cells (2). Serum IL-2 levels are associated with multiple cardiovascular diseases such as coronary artery disease and cardiac arrhythmias (CAs) (3).

Atrial fibrillation (AF) and ventricular tachycardia/ventricular fibrillation (VT/VF) are the two main types of CAs that are highly associated with the level of serum IL-2 (4–6). IL-2 has also been associated with the recurrence of AF in patients undergoing catheter ablation (5). In addition, high-dose IL-2 therapy has been significantly associated with cardiac toxicities in patients with cancer, often resulting in lethal arrhythmias (6–8). However, the underlying mechanism of IL-2 in the pathogenesis of CAs still remains unclear.

Dysfunction of ion channels plays an important role in the etiology of CAs (9). Family-based genetic studies have revealed that both loss-of-function and gain-of-function mutations of genes encoding ion channels can lead to CAs including AF (10) as well as VT/VF (11, 12). IL-2 has been confirmed to regulate the expression of the voltage-gated sodium channel (Nav1.5) complex including sodium voltage-gated channel alpha subunit 3 (SCN3A), sodium voltage-gated channel beta subunit 3 (SCN3B), and sodium voltage-gated channel alpha subunit 4 (SCN4A). In particular, the expression of SCN3B has been reported to be upregulated by IL-2 via the p53 pathway. Overexpression of SCN3B has been found to play a gain-of-function-like effect, increase the fast inward cardiac sodium current-*I*_Na_, and possibly cause CAs (13). However, the reasons behind the increased serum IL-2 levels and the potential regulatory factor(s) related to the expression of IL-2 in patients with CAs are still unknown.

The expression of *miR-190a-5p* is altered in response to hypoxia and circulating *miR-190a-5p* is a possible biomarker of chronic heart failure (14). *IL-2* has been observed to be possibly regulated by *miR-190-5p*, as per TargetScan 7.2 (15). These reports prompted us to evaluate the effect of *miR-190a-5p* on IL-2 expression in the progress of CAs. This study aimed to: (1) evaluate the expression of peripheral blood serum IL-2 in patients with AF, (2) verify the correlation between IL-2 and *miR-190-5p* expression in myocardial tissues of patients with AF, (3) clarify the role of *miR-190a-5p* in regulating the abnormal electrical activity of sodium current density in SCN3B, and (4) reveal the possibility of *miR-190a-5p* being a protective factor against the progress of CAs through negative regulation of IL-2.

## Materials and Methods

### Ethics Statement

This study involving human participants was reviewed and approved by the appropriate Local Institutional Review Board on human subject research at Huazhong University of Science and Technology and the First Affiliated Hospital of Xiamen University and also conformed to the guidelines set forth by the Declaration of Helsinki. The patients/participants (legal guardian/next of kin) provided a written informed consent to participate in this study. This study was conducted in accordance with the International Ethical Guidelines for Biomedical Research Involving Human Subjects (CIOMS).

### Sample Collection

Peripheral blood serum samples of 61 clinical subjects, including patients with AF (*n* = 34) (Table 1) and normal controls (*n* = 27) with normal ECG, were collected from Tongji Hospital Affiliated to Huazhong University of Science and Technology. Human cardiac tissues from patients with AF (*n* = 4) and those without AF (non-AF) (*n* = 4) were collected from the First Affiliated Hospital of Xiamen University. All the patients with AF were diagnosed by visual inspection of the ECG.

**Table 1:** Clinical information of patients with AF.

**ID No**.	**Review or not**	**Hospitalized No**.	**Gender**	**Related clinical symptoms**
160101	No	1254639	Male	Permanent AF, New York Heart Function Classification (NYHA) class III, chronic obstructive emphysema with acute exacerbation, coronary atherosclerotic heart disease, arrhythmia.
160112	No	1254948	Male	AF, wide QRS complex tachycardia (ventricular tachycardia), acute coronary syndrome, hypertension grade 3, type 2 diabetes, cerebral infarction, liver insufficiency, renal insufficiency.
160118	No	1254970	Male	AF, coronary atherosclerotic heart disease, NYHA class III.
160122	No	1254513	Female	AF, rheumatic heart disease, rheumatic mitral stenosis with insufficiency, aortic regurgitation, NYHA class III, type 2 diabetes, coronary atherosclerosis, hypertension grade 3 (extremely high risk), anemia.
160125	No	1254710	Female	AF, coronary heart disease, acute coronary syndrome, NYHA class II, hypertension grade 3(extremely high risk), herpes.
160128	No	1255356	Female	AF, coronary heart disease, angina pectoris, arrhythmia, post-percutaneous coronary intervention (PCI), NYHA class III, hypertension level 3.
160130	No	1255576	Female	AF, coronary heart disease, hypertension level 2.
160155	No	1255273	Male	AF, coronary heart disease, arrhythmia, NYHA class II, hypertension, digestive system disease.
160160	No	1256003	Male	AF, heart valve disease, moderate mitral regurgitation, coronary heart disease, arrhythmia, NYHA class III, connective tissue disease, lung infection.
160170	No	1255921	Male	AF, arrhythmia, paroxysmal supraventricular tachycardia.
160200	No	1255236	Female	Persistent AF, hypertension grade 3, arrhythmia, post implantation of permanent cardiac pacemaker.
160208	Yes (160168)	1256341	Male	AF, hypertension grade 3, hypertensive heart disease, coronary atherosclerotic heart disease, prior myocardial infarction, NYHA class II, arrhythmia, chronic bronchitis.
160226	No	1257212	Female	AF, coronary heart disease, valvular heart disease, moderate tricuspid regurgitation, NYHA class III, hypertension grade 3, type 2 diabetes, renal artery stenosis.
160232	No	1256854	Male	AF, arrhythmia, coronary heart disease, schistosomiasis liver disease, splenectomy, hepatitis B.
160234	No	1257535	Male	AF, arrhythmia, hypertension grade 3, type 2 diabetes, hyperuricemia, hyperlipidemia.
160262	No	1258107	Male	AF, paroxysmal supraventricular tachycardia, NYHA class II.
160269	No	1257444	Male	AF, arrhythmia.
160271	No	1258363	Male	AF with long intervals, coronary atherosclerotic heart disease, NYHA class III, emphysema, lung infection, hypertension grade 3 (very high risk).
160281	No	1258826	Male	Paroxysmal AF, hypertension.
160285	No	1258637	Male	AF, arrhythmia, ventricular premature beats, bronchial asthma.
160292	No	1258209	Male	AF, cerebral infarction, coronary heart disease, pacemaker implantation, type 2 diabetes.
160297	No	1257856	Female	AF, coronary heart disease, hypertension grade 3 (very high-risk group), type 2 diabetes, peripheral neuropathy.
160309	No	1258911	Female	AF, arrhythmia, paroxysmal supraventricular tachycardia, frequent premature ventricular, hypertension grade 3.
160449	Yes (160271)	1264487	Male	AF, coronary atherosclerotic heart disease, NYHA class III, chronic obstructive pulmonary disease, hypertension grade 3 (extremely high risk).
160015P1	No	1253229	Male	AF, coronary heart disease, arrhythmia, lung infection.
160024P1	No	1252735	Female	AF, coronary heart disease, post- PCI, arrhythmia, NYHA class III, radiofrequency ablation of atrial flutter.
160027P1	No	1253389	Female	AF, hypertension grade 3, hypertensive heart disease, NYHA class III, coronary atherosclerotic heart disease, chronic renal insufficiency, sequelae of cerebral infarction.
160035P1	No	1253856	Male	Paroxysmal AF, arrhythmia, hypertension grade 3.
160043P1	No	1253789	Male	AF, arrhythmia, paroxysmal atrial flutter, post cardiac radiofrequency ablation, hypertension grade 3 (extremely high-risk).
160049P1	No	1253907	Male	AF, coronary atherosclerotic heart disease, unstable angina pectoris, NYHA class II, hypertension grade 3, sick sinus syndrome, after permanent cardiac single-chamber pacemaker implantation, type 2 diabetes.
160050P1	No	1253643	Female	AF, coronary heart disease, arrhythmia, NYHA class II.
160056P1	No	1254269	Male	AF, hypertension grade 3, coronary heart disease, prior myocardial infarction (2013), ventricular aneurysm, post-PCI, significant sinus bradycardia, permanent cardiac pacemaker implantation.
160081P1	No	1254498	Female	AF, coronary atherosclerotic heart disease, prior myocardial infarction, post-PCI, NYHA class IV, hypertension grade 3, senile dementia.
160088P1	No	1254245	Female	Paroxysmal AF, arrhythmia, post radiofrequency ablation, hypertension, coronary atherosclerosis.
160092P1	No	1254441	Female	Paroxysmal AF, hypertension grade 3, type 2 diabetes, chronic renal insufficiency, hypothyroidism, ankylosing spondylitis, lung infection.

### Bioinformatics-Based Prediction

TargetScan 7.2 (http://www.targetscan.org/vert72/) was used to predict the potential microRNAs [miRNA(s)] directly regulating the downstream target gene *IL-2* and the specific binding site(s) of miRNA(s) (or seed sequences) in the 3'-untranslated region (UTR) of *IL-2* (Table 2, marked in blue).

**Table 2:** Binding sites (blue) of *miR-181* and *miR-191* in the 3'-untranslated region (UTR) of interleukin-2 (*IL-2*).

**Positions in 3'-UTR and miRNAs**	**Predicted consequential pairing of target region (top) and miRNA (bottom)**	**Site type**	**Context++ score**	**Context++ score percentile**	**Weighted context++ score**	**Conserved branch length**	**P_**CT**_**
74–81 *miR-181a-5p*	5'...UUUUAUAUUUAUUGUUGAAUGUA... 3' UGAGUGGCUGUCGCAACUUACAA	8mer	−0.46	99	−0.46	1.034	<0.1
215–221 *miR-181a-5p*	5'...UAUUUAUUAUUAUGUUGAAUGUU... 3' UGAGUGGCUGUCGCAACUUACAA	7mer-m8	−0.25	95	−0.25	0.284	<0.1
74–81 *miR-181b-5p*	5'...UUUUAUAUUUAUUGUUGAAUGUA... 3' UGGGUGGCUGUCGUUACUUACAA	8mer	−0.44	98	−0.44	1.034	<0.1
215–221 *miR-181b-5p*	5'...UAUUUAUUAUUAUGUUGAAUGUU.. . 3' UGGGUGGCUGUCGUUACUUACAA	7mer-m8	−0.23	94	−0.23	0.284	<0.1
74–81 *miR-181c-5p*	5'...UUUUAUAUUUAUUGUUGAAUGUA... 3' UGAGUGGCUGUCCAACUUACAA	8mer	−0.46	99	−0.46	1.034	<0.1
215–221 *miR-181c-5p*	5'...UAUUUAUUAUUAUGUUGAAUGUU... 3' UGAGUGGCUGUCCAACUUACAA	7mer-m8	−0.25	95	−0.25	0.284	<0.1
74–81 *miR-181d-5p*	5'...UUUUAUAUUUAUUGUUGAAUGUA... 3' UGGGUGGCUGUUGUUACUUACAA	8mer	−0.44	98	−0.44	1.034	<0.1
215–221 *miR-181d-5p*	5'...UAUUUAUUAUUAUGUUGAAUGUU.. . 3' UGGGUGGCUGUUGUUACUUACAA	7mer-m8	−0.23	94	−0.23	0.284	<0.1
24–31 *miR-190a-5p*	5'...UGCUUCCCACUUAAAACAUAUCA... 3' UGGAUUAUAUAGUUUGUAUAGU	8mer	−0.68	99	−0.68	0.100	<0.1
24–31 *miR-190b*	5'...UGCUUCCCACUUAAAACAUAUCA... 3' UUGGGUUAUAGUUUGUAUAGU	8mer	−0.69	99	−0.69	0.100	<0.1

### Cell Lines and miRNAs

AC16 cell line is a type of human myocardial cells that is commonly used to study the cardiovascular diseases *in vitro*. In this study, human Raji cell line was used to verify the results acquired from AC16 cells. AC16 and Raji cell lines were purchased from American Type Culture Collection (ATCC, Rockville, Maryland, USA). HEK293 and HEK293 cells stably overexpressing SCN5A (HEK293/Nav1.5) were available in our own laboratory (16). AC16, HEK293, and HEK293/Nav1.5 cells were cultured in Dulbecco's Modified Eagle's Medium (Gibco Life Technologies, Grand Island, Nebraska, USA) and Raji cells were cultured in Roswell Park Memorial Institute (RPMI) 1640 medium at 37°C with 5% carbon dioxide (CO_2_).

All the miRNA mimics, inhibitors, and negative controls were purchased from RiboBio Corporation Limited (Guangzhou, China).

### Double Luciferase Reporter Assay

The PCR product (282 bp) of the 3'-UTR of *IL-2*, containing the predicted binding sites of *miR-181* (*miR-181a-5p, miR-181b-5p, miR-181c-5p*, and *miR-181d-5p*) and *miR-190* (*miR-190a-5p* and *miR-190b-5p*) families, was subcloned into the empty vector of pMIR-REPORT (Applied Biosystems, Foster City, California, USA), named *IL-2*-pMIR-REPORT-WT, to screen for the effective miRNA(s) targeting *IL-2*. Subsequently, the binding site was mutated (*IL-2*-pMIR-REPORT-MU) by site-directed mutagenesis to further confirm the binding site of *miR-190a-5p*.

To screen for miRNA(s) that targeted *IL-2*, HEK293 cells, cultured at 37°C with 5% CO_2_ for 24 h, were co-transfected with: (1) pMIR-REPORT + miR-NC, (2) *IL-2*-pMIR-REPORT-WT + miR-NC, (3) pMIR-REPORT + miRNA libraries (*miR-181* or *miR-190*), and (4) *IL-2*-pMIR-REPORT-WT + miRNA libraries (*miR-181* or *miR-190*).

To further confirm the relationship between *miR-190a-5p* and IL-2, HEK293 cells, at 37°C with 5% CO_2_ for 24 h, were co-transfected with: (1) pMIR-REPORT + inhibitor-NC, (2) *IL-2*-pMIR-REPORT-WT + inhibitor-NC, (3) pMIR-REPORT + *miR-190a-5p* inhibitor, (4) *IL-2*-pMIR-REPORT-WT + *miR-190a-5p* inhibitor, (5) *IL-2*-pMIR-REPORT-MU + miR-NC, and (6) *IL-2*-pMIR-REPORT-MU + *miR-190a-5p*. After 48 h, luciferase signal intensity was measured using the Dual-Luciferase Reporter Assay System (PR-E1910, Promega, Madison, Wisconsin, USA) on GloMax 20/20 (Promega, Madison, Wisconsin, USA). Relative luciferase intensity was normalized to Renilla luciferase activity.

### Quantitative Real-Time-PCR (qRT-PCR)

AC16 cells and Raji cells, cultured at 37°C with 5% CO_2_ for 24 h, were transfected with *miR-190a-5p* mimic/inhibitor or miR-NC/inhibitor-NC (RiboBio Corporation Limited, Guangzhou, China) for 48 h. The cells were then lysed using RNAiso Plus (Takara Biomedical Technology, Dalian, China). Total RNA was extracted and reverse transcribed into complementary DNA (cDNA) using the M-MLV Reverse Transcription Kit (Vazyme, Nanjing, China) in accordance with the instructions of the manufacturer. qRT-PCR was conducted with AceQ qPCR SYBR Green Master Mix (Q141-02/03, Vazyme, Nanjing, China) on the ABI StepOnePlus™ Real-Time PCR System. The primer sequence used was as follows: *IL-2*: 5′-AGGCCACAGAACTGAAAC-3′ (Forward), 5′-TTACGTTGATATTGCTGATTA-3′ (Reverse); *SCN3B*: 5′-GCCTTCAATAGATTGTTTCCCCT-3′ (Forward), 5′-CTCGGGCCTGTAGAACCAT-3′ (Reverse); and glyceraldehyde 3-phosphate dehydrogenase (*GAPDH*): 5′-GGAGCGAGATCCCTCCAAAAT-3′ (Forward), 5′-GGCT GTTGTCATACTTCTCATGG-3′ (Reverse). For reverse transcription and qRT-PCR of *miR-190a-5p*, the Bulge-Loop™ qRT-PCR primer sets of *miR-190a-5p* and *U6* were used (RiboBio Corporation Limited, Guangzhou, China).

### Enzyme-Linked Immunosorbent Assay

The concentration of IL-2 in peripheral blood serum of clinical samples was detected by ELISA using the Chemiluminescent Immunoassay Kit for IL-2 (SCA073Hu, Houston, Texas, USA) according to the instructions of the manufacturer.

AC16 and Raji cells were plated in 6-well plates at 37°C with 5% CO_2_ for 24 h and treated with *miR-190a-5p* mimic/miR-NC or *miR-190a-5p* inhibitor/inhibitor-NC for 48 h. Thereafter, the culture supernatant was collected and concentration of IL-2 was determined by ELISA.

### Whole-Cell Patch-Clamp Experiments

Nav1.5 is a sodium channel subunit that generates sodium current in the heart. Sodium current is commonly analyzed by patch-clamping of HEK293 cells (17). Change in sodium current density in SCN3B was detected by whole-cell patch-clamp technique using HEK293/Nav1.5 cells. After being cultured for 24 h, 2 μg pEGFP-N1/pEGFP-N1-SCN3B (13), *miR-190a-5p* mimic/miR-NC, or *miR-190a-5p* inhibitor/inhibitor-NC was co-transfected at 70–80% confluence. After 48 h, green fluorescent protein (GFP)-positive cells were selected for electrophysiological studies according to the standardized experimental procedures (17).

### Fluorescence *in situ* Hybridization

Human cardiac tissues from patients with AF and from non-AF controls (Table 3) were fixed with 4% paraformaldehyde for fluorescence *in situ* hybridization (FISH). Briefly, paraffin-embedded sections of cardiac tissues were subjected to high-pressure antigen retrieval in citrate buffer (pH 6.0). Sections were blocked in 5% bovine serum albumin (BSA), incubated with IL-2 primary antibodies (NBP2-16948, NOVUS, Colorado, USA), and then incubated with Cy3-conjugated goat antirabbit immunoglobulin G (IgG) (H + L) (BA1032; Boster Biological Technology Corporation Ltd., Wuhan, China). Next, the sections were incubated with *miR-190a-5p* Dig-labeled antisense probe (5′-ACCUAAUAUAUCAAACAUAUCA-3′) and then treated with Alexa Fluor 488-labeled goat antirabbit IgG (H + L) (A0423, Beyotime, Shanghai, China). Finally, nuclei were stained with 4′,6-diamidino-2-phenylindole (DAPI) (1:2,000). The PANNORAMIC MIDI II (3DHISTECH, Budapest, Hungary, UK) was used to detect images of the slides.

**Table 3:** Clinical information of subjects for fluorescence *in situ* hybridization (FISH).

**Patient ID**	**Hospitalized no**.	**Sex**	**Age (years)**	**AF**	**Other cardiovascular diseases**	**Tissue source**
Patient 1	552954	Female	53	Yes	Heart valve disease, heart enlargement	Tendons from valvula bicuspidalis
Patient 2	554552	Female	65	Yes	Heart valve disease, cardiac insufficiency	Right auricle
Patient 3	442617	Female	59	Yes	Heart valve disease, rheumatic heart disease	Right auricle
Patient 4	568001	Male	39	Yes	Heart valve disease, rheumatic heart disease, heart enlargement, cardiac insufficiency	Left auricle
Control 1	553053	Male	0.4	No	Congenital heart disease, ventricular septal defect	Right auricle
Control 2	556812	Male	4	No	ventricular septal defect	Right auricle
Control 3	558147	Male	0.5	No	Congenital heart disease, ventricular septal defect	Right auricle
Control 4	561361	Male	4	No	Congenital heart disease, ventricular septal defect	Right auricle

### Data Analysis

Data are presented as the mean ± SEM. The GraphPad Prism version 6 (San Diego, California, USA) was used for statistical analysis. The one-way ANOVA and the Student's two-tailed *t*-tests were used for multiple-group comparisons and between-group comparisons, respectively. Statistically significant difference was considered at *p* < 0.05 (^*^p < 0.05, ^**^p < 0.01).

## Results

### Serum IL-2 Concentration Increased in Patients With AF

**Figure 1:**
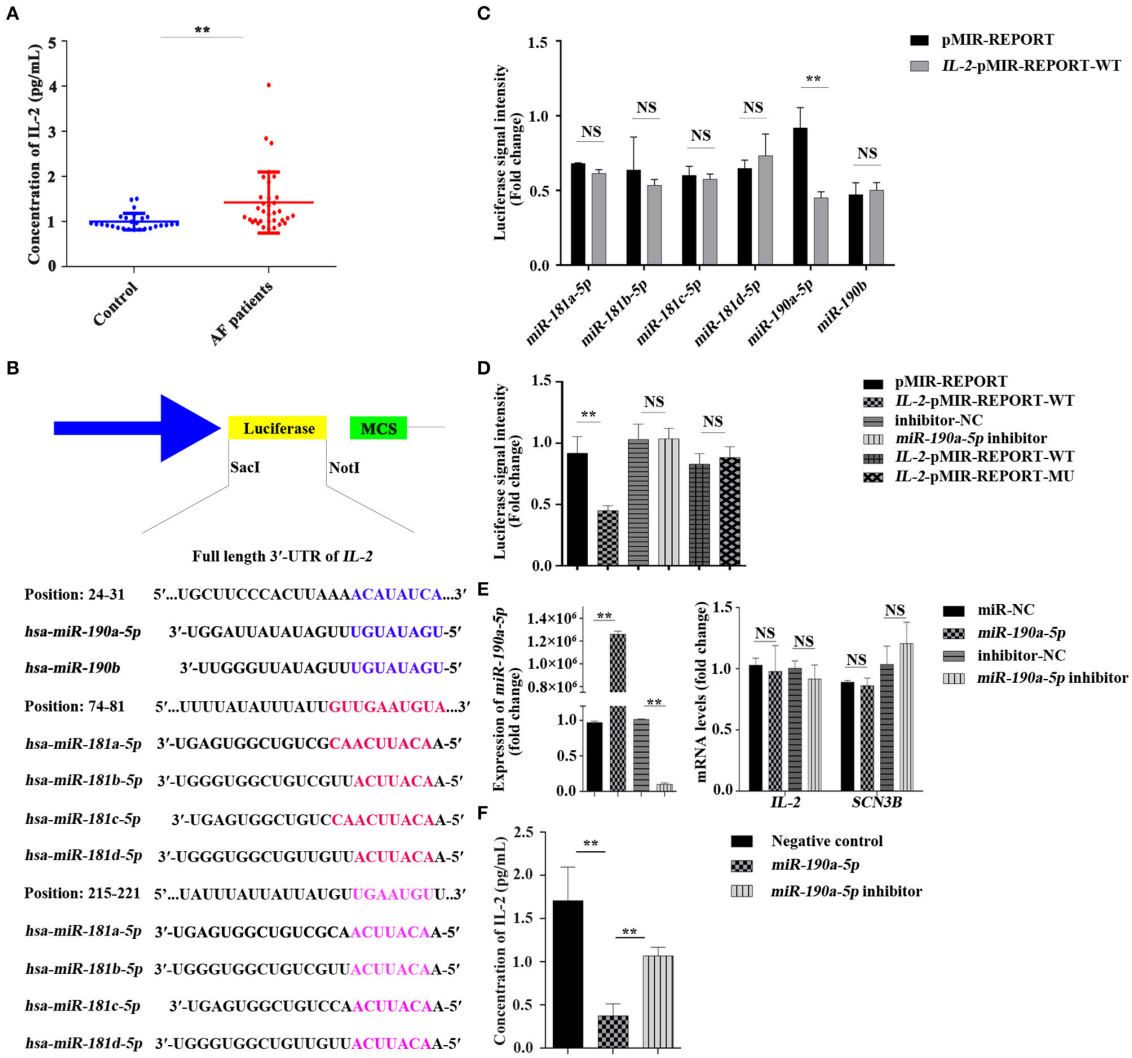
Concentration of interleukin-2 (IL-2) in the peripheral blood serum of patients with atrial fibrillation (AF) was increased and IL-2 was negatively regulated by *miR-190a-5p*. **(A)** Level of IL-2 was increased in the peripheral blood serum of patients with AF than that of the normal control group (**p < 0.01 *vs*. control group). **(B)** The binding sites of *miR-181* and *miR-190* families in the 3'-untranslated region (UTR) of *IL-2*. **(C,D)** Relative luciferase signal intensity in HEK293 cells (NS, no significant difference, **p < 0.01 *vs*. pMIR-REPORT). **(E)** Relative expression of *miR-190a-5p, IL-2*, and sodium voltage-gated channel beta subunit 3 (*SCN3B*) in AC16 cells transfected with *miR-190a-5p* mimic/inhibitor or miR-NC/inhibitor-negative control (NC) (**p < 0.01 *vs*. miR-NC, NS *vs*. inhibitor-NC). **(F)** Concentration of IL-2 in culture medium of AC16 cells treated with *miR-190a-5p* mimic/inhibitor or Negative control (NC) (**p < 0.01 *vs*. NC, **p < 0.01 *vs*. *miR-190a-5p*). Data are represented as means ± SEM.

Many inflammatory markers, including IL-2, are associated with the presence or outcome of AF (18). To confirm the relationship between IL-2 and AF, an ELISA was performed to detect the change in IL-2 concentration in the peripheral blood serum of patients with AF. Results demonstrated that the level of IL-2 in the peripheral blood serum of patients with AF increased by 42.38% compared to that in the normal control group (0.9980 ± 0.03558 *vs*. 1.4210 ± 0.1163 pg ml^−1^, ^**^p < 0.01, Figure 1A), suggesting that the increase in IL-2 levels in the peripheral blood serum of patients with AF may be related to the inflammatory changes caused by AF.

### Interleukin-2 Was Negatively Regulated by *miR-190a-5p*

Online bioinformatics-based prediction indicated that *miR-180* or *miR-190* families may target the downstream *IL-2* (Figure 1B) either alone or together. Results of the double luciferase reporter assay confirmed that luciferase signal intensity from the vector carrying the *IL-2* wild-type reporter gene (*IL-2*-pMIR-REPORT-WT) was notably decreased by 50.92% after transfection with *miR-190a-5p* mimic, though not with other miRNAs, compared to that of pMIR-REPORT (^**^p < 0.01, Figure 1C), whereas the *miR-190a-5p* inhibitor passivated this effect (*p* > 0.5, Figure 1D). The binding site (5′-ACAUAUCA-3′) was mutated (5′-GTGGCGTC-3′) to further verify the targeting of *miR-190a-5p* to *IL-2*. Luciferase signal intensity from the vector carrying the *IL-2* mutant reporter gene (*IL-2*-pMIR-REPORT-MU) was not significantly different from that carrying *IL-2*-pMIR-REPORT-WT (*p* > 0.5, Figure 1D).

AC16 and Raji cells, seeded in 6-well plates at 37°C with 5% CO_2_ for 24 h, were transfected with *miR-190a-5p* mimic/inhibitor or miR-NC/inhibitor-NC for 48 h. The adherent cells were collected to detect the expression of *miR-190a-5p, IL-2*, and *SCN3B* by qRT-PCR and the culture medium was retained for detection of IL-2 concentration by ELISA. Results of qRT-PCR revealed that, compared to miR-NC, *miR-190a-5p* was overexpressed by 1298127.275-fold in AC16 cells (^**^p < 0.01, Figure 1E) and 422.22-fold in Raji cells (^**^p < 0.01, Supplementary Figure 1A). The expression of *miR-190a-5p* was remarkably downregulated by 90.13% when treated with *miR-190a-5p* inhibitor in AC16 cells than when treated with inhibitor-NC (^**^p < 0.01, Figure 1E); it did not change significantly after transfection with either inhibitor-NC or *miR-190a-5p* inhibitor in Raji cells (Supplementary Figure 1A); the differential effects of *miR-190a-5p* inhibitor may be due to the differences in cell types. The expression of *IL-2* did not change when cells (both AC16 and Raji cells) were treated with *miR-190a-5p* mimics or inhibitors (Figure 1E and Supplementary Figure 1B) and the expression of *SCN3B*, after transfection with *miR-190a-5p* mimic or inhibitor, was similar to that of *IL-2* in AC16 cells (Figure 1E). In AC16 cells, the concentration of IL-2 in the medium supernatant was decreased by 78.11% when treated with *miR-190a-5p* (1.707 ± 0.3904 *vs*. 0.3737 ± 0.1397 pg ml^−1^) (^**^p < 0.01, Figure 1F), whereas treatment with *miR-190a-5p* inhibitor increased the concentration of IL-2 by 65.01% (0.3737 ± 0.1397 *vs*. 1.068 ± 0.1001 pg ml^−1^, ^**^p < 0.01, Figure 1F). In Raji cells, the concentration of IL-2 in the medium supernatant decreased by 11.73% when treated with *miR-190a-5p* (163.7 ± 4.070 *vs*. 144.5 ± 1.703 pg ml^−1^, ^**^p < 0.01, Supplementary Figure 1C); however, treatment with *miR-190a-5p* inhibitor did not affect the secretion of IL-2 (Supplementary Figure 1C). These data collectively indicated that *miR-190a-5p* may affect the expression of IL-2 by directly targeting the 3′-UTR binding site of *IL-2*.

### *miR-190a-5p* Reversed the Increased Sodium Current (*I*_Na_) Caused by Increased Endogenous IL-2

**Figure 2:**
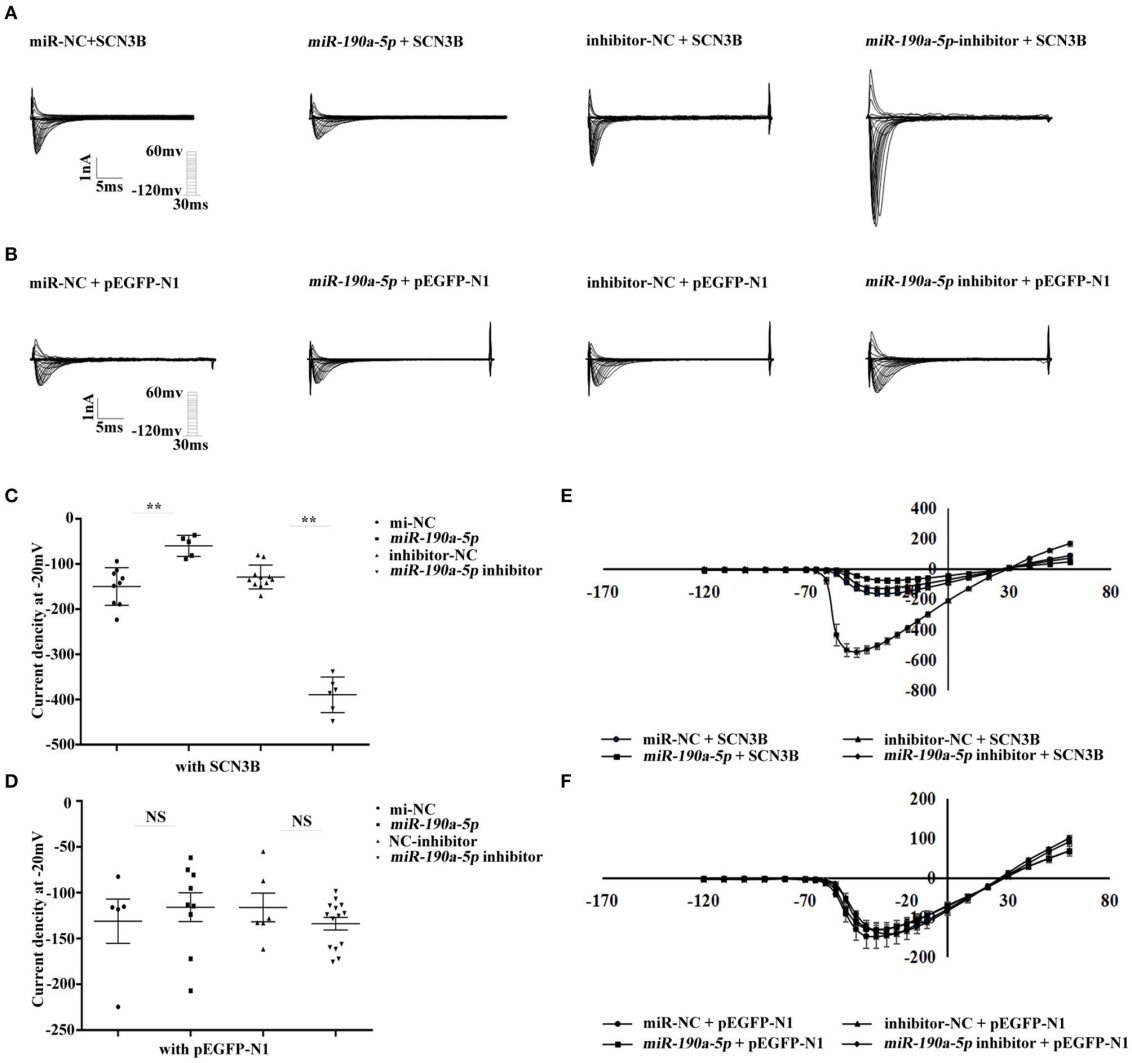
*miR-190a-5p* reduced the sodium current density of SCN3B. **(A,C,E)** Representative traces of sodium currents, histogram of sodium current densities at −20 mV, and IV relation for peak sodium current Nav1.5 from HEK293/Nav1.5 cells, respectively, transfected with: (1) miR-NC + SCN3B, (2) *miR-190a-5p* + SCN3B, (3) inhibitor-NC + SCN3B, and (4) *miR-190a-5p inhibitor* + SCN3B (**p < 0.01 *vs*. miR-NC, **p < 0.01 *vs*. inhibitor-NC). Data are represented as means ± SEM. **(B,D,F)** Representative traces of sodium currents, histogram of sodium current densities at −20 mV, and IV relation for peak sodium current Nav1.5 from HEK293/Nav1.5 cells, respectively, transfected with: (1) miR-NC + pEGFP-N1, (2) *miR-190a-5p* + pEGFP-N1, (3) inhibitor-NC + pEGFP-N1, and (4) *miR-190a-5p inhibitor* + pEGFP-N1 (NS, no significant difference *vs*. miR-NC, NS *vs*. inhibitor-NC). Data are represented as means ± SEM.

Changes in sodium current were analyzed by whole-cell patch clamping in HEK293/Nav1.5 cells. In order to investigate whether *miR-190a-5p* affected the sodium current density in SCN3B at the endogenous level, pEGFP-N1/pEGFP-N1-SCN3B, *miR-190a-5p* mimic/miR-NC, or *miR-190a-5p* inhibitor/inhibitor-NC was co-transfected in HEK293/Nav1.5 cells. After treatment with *miR-190a-5p*, the sodium current density (expressed as the normalized peak current relative to the battery capacitance, pA/pF) decreased by 59.98% (^**^p < 0.01) over the entire test potential range; it increased 2.04-fold in the cells transfected with *miR-190a-5p* inhibitor (^**^p < 0.01, Figures 2A,C,E). miR-NC or inhibitor-NC failed to affect the sodium currents (Figures 2B,D,F). The results suggested that *miR-190a-5p* may downregulate the endogenous expression of IL-2, which induces abnormal sodium channel current of SCN3B, eventually accelerating the pathogenesis of CAs.

### *miR-190a-5p* Decreased While IL-2 Increased in Human AF Cardiac Tissues

**Figure 3:**
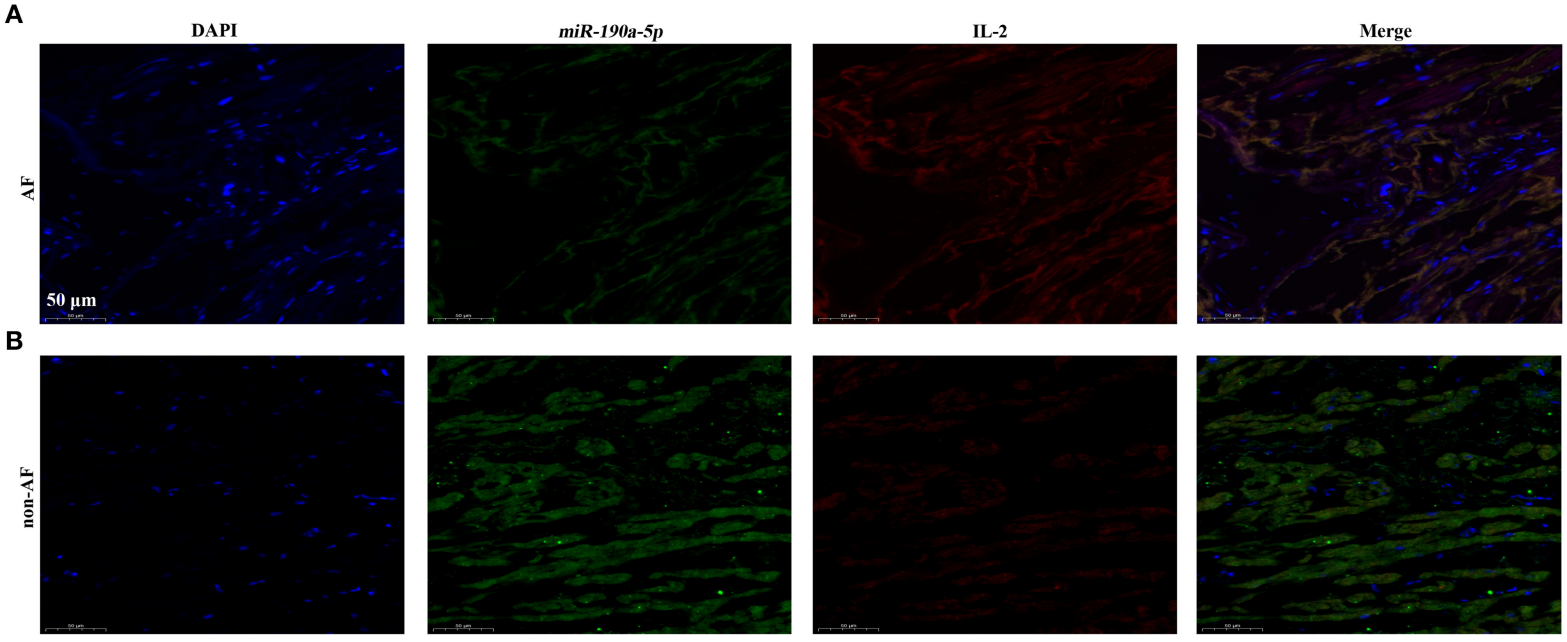
In cardiac tissues of patients with AF, *miR-190a-5p* was downregulated while IL-2 was upregulated. **(A)** Immunofluorescence co-staining of *miR-190a-5p* and IL-2 in human cardiac tissues of patients with AF. **(B)** Immunofluorescence co-staining of *miR-190a-5p* and IL-2 in human cardiac tissues from non-AF control. Scale bar = 50 μm, *n* = 4 per group.

Interleukin-2 has been verified to be a direct target of *miR-190a-5p* at the cellular level. However, co-localization of *miR-190a-5p* and IL-2 needed to be confirmed in human AF cardiac tissues. Results of co-immunofluorescence of *miR-190a-5p* and IL-2 revealed that *miR-190a-5p* (green) was downregulated, while IL-2 (red) was upregulated in the AF group compared to that in the non-AF group (Figure 3), suggesting the negative regulatory relationship between *miR-190a-5p* and *IL-2*.

## Discussion

In this study, the level of peripheral blood serum IL-2 was found to be increased by 42.38% in patients with AF compared to that in normal controls (^**^p < 0.01, Figure 1A); *miR-190a-5p* was downregulated, while IL-2 was upregulated in human AF cardiac tissues compared to that in non-AF controls (Figure 3). Furthermore, after treatment with *miR-190a-5p*, the sodium current density decreased by 59.98% over the entire test potential range; it increased 2.04-fold in the cells transfected with *miR-190a-5p* inhibitor (Figures 2A,C,E). Based on such evidence, we confirmed for the first time that *miR-190a-5p* may play a role in the causation of CAs by negatively regulating IL-2 and reducing the density of sodium current peaks produced by SCN3B.

*miR-190* (gene ID^*^ 406965), including two major mature forms of *miR-190-5p* and *miR-190-3p*, is located near the long arm of chromosome 15 (15q22.2) (19). The biological function of *miR-190-3p* has rarely been investigated. Jin et al. had confirmed that *miR-190-3p* participates in the formation of glioma *via* the prostate androgen-regulated transcript 1 (PART1)/*miR-190a-3p*/phosphatase and tensin homolog deleted on chromosome ten (PTEN)/phosphatidylinositol-3-kinase (PI3K)/protein kinase B (AKT) pathway (20). Experimental evidence had indicated *miR-190-5p* to be associated with various human diseases such as diabetic neuropathic pain (21), breast cancer (22), Parkinson's disease (23), pulmonary arterial hypertension (24), and diabetes mellitus (25). However, whether *miR-190a-5p* is involved in the occurrence of CAs is not yet clear and the specific molecular mechanism needs to be further explored.

Levels of IL-2 have been associated with reduced incidence of postoperative AF and supraventricular tachycardia (6, 26). In this *in*-*vivo* study, the level of IL-2 was remarkably increased in the peripheral blood of patients with AF; moreover, *miR-190a-5p* was downregulated, while IL-2 was upregulated in human AF cardiac tissues. In *in*-*vitro* experiments, *miR-190a-5p* did not affect the transcription level of IL-2 in AC16 and Raji cells, whereas it significantly reduced both the secretion of IL-2 in the medium supernatant of both kinds of cells and the *I*_Na_ of SCN3B in HEK293/Nav1.5 cells, which, in turn, was aggravated by inhibition of *miR-190a-5p*. The results, therefore, suggested that *miR-190a-5p* might play an important role in the progression of CAs by negatively regulating IL-2.

Furthermore, *miR-190a-5p* was shown to decrease the sodium current intensity of SCN3B, while its inhibition significantly increased the same. Olesen et al. had demonstrated that R6K, L10P, and M161T in *SCN3B* (NM_018400) were associated with early lone AF (27). Valdivia et al. had reported a V54G mutation in *SCN3B* of a patient diagnosed with idiopathic ventricular fibrillation (IVF) and indicated this mutation to cause “loss-of-function” of *SCN3B* by decreasing *I*_Na_ by 70 and 90% in HEK293 and primate fibroblastoid COS cells, respectively (28). In Japan, V110I in *SCN3B* is a relatively common cause of *SCN5A*-negative Brugada syndrome, which eventually results in a decrease in sodium current due to the lack of cell surface expression of Nav1.5 (29). This evidence supported the fact that *SCN3B* is closely related to CAs. Further, IL-2 increases the current intensity of SCN3B via the p53 pathway (13). Therefore, *miR-190a-5p* may partially rescue the abnormal cardiac electrical activity of patients with CAs, to some extent, by reducing the expression of IL-2. Furthermore, we speculated that there may be an important role of other miRNA(s) or genes in the pathological process of CAs via regulation of the current intensity of SCN3B or the stability of ion channels; this mechanism needs to be further explored and studied in future.

This study has some limitations. *miR-190a-5p* has been suggested to possibly play a role in protecting against CAs; however, the relationship between *miR-190a-5p* and *IL-2* did not display one-to-one stoichiometry. Furthermore, one specific miRNA may simultaneously target multiple genes. The existing results did not exclude whether *miR-190a-5p* targets one or more other genes concurrently while exerting a protective role in the pathological process of CAs. The role of *miR-190a-5p* and other target genes, besides IL-2, in the pathological progress of CAs and the specific molecular mechanism, would need further exploration. Although both *miR-190a-5p* and IL-2 are closely associated with CAs, whether the expression of IL-2 is affected by *miR-190a-5p* in the long run and the exact role of *miR-190a-5p* and IL-2 in patients with CAs would require further evidence in future prospective clinical studies. Considering that CAs are divided into many types, whether *miR-190a-5p* and IL-2 play identical roles in many other CAs remains to be addressed. Since the sequence of *miR-190a-5p* binding to *IL-2* is not well-conserved across humans, mice, and rats, it cannot be verified by animal model experiments. Some other miRNAs, which are higher more conserved across mammalian species, might also play an important role in affecting the expression of IL-2 in AF; therefore, future studies would need to be performed to discover the potential candidates. In this study, only eight tissue samples (auricle or valvula bicuspidalis) were collected, due to the major challenge of collecting human cardiac tissue samples. In addition, sex-related differences exist in a wide variety of CAs (30–32). Therefore, the relatively low volume of data in human AF study was the major limitation of this study. In future, more cardiac tissue samples from patients with AF and from non-AF controls would need to be collected to further confirm the accuracy of FISH results. Expansion of the clinical sample size would be necessary in future studies.

In conclusion, this study confirmed that *miR-190a-5p* may partially block the abnormal electrical activity of SCN3B in the progression of CAs and the possible pathway could be via *miR-190a-5p*/IL-2/SCN3B. Although the specific mechanism needs to be confirmed in further experiments, *miR-190a-5p* may still be regarded as a potential clinical target for CAs.

## Data Availability Statement

The original contributions presented in the study are included in the article/Supplementary Material, further inquiries can be directed to the corresponding author/s.

## Ethics Statement

The studies involving human participants were reviewed and approved by the appropriate Local Institutional Review Boards on human subject research at Huazhong University of Science and Technology and the First Affiliated Hospital of Xiamen University and also conformed to the guidelines set forth by the Declaration of Helsinki. The patients/participants (legal guardian/next of kin) provided written informed consent to participate in this research. The project was conducted in accordance with the International Ethical Guidelines for Biomedical Research Involving Human Subjects (CIOMS). Written informed consent to participate in this study was provided by the participants' legal guardian/next of kin.

## Author Contributions

QL, ZZ, SC, and YZ performed the experimental work. ZZ, MW, MZ, CY, XW, YC, and ZH provided the clinical samples and assisted in analysis. YZ, QL, ZZ, DJ, DD, YH, ZH, and XT analyzed the data and provided advice. YZ, ZC, and XT designed the experiments. QL, YZ, and XT wrote the manuscript, obtained funding for this project, directed, and supervised the study. All authors have read and agreed to the published version of the manuscript.

## Funding

This study was funded by grants from the National Natural Science Foundation of China (Grant Nos. 81700300, 82000321, 81700302, 81770652, and 81800296) and the Natural Science Foundation of Hubei Province (2017CFB322).

## Conflict of Interest

The authors declare that the research was conducted in the absence of any commercial or financial relationships that could be construed as a potential conflict of interest. The reviewer LG declared a shared affiliation with one of the authors, QL to the handling editor at time of review.

## Publisher's Note

All claims expressed in this article are solely those of the authors and do not necessarily represent those of their affiliated organizations, or those of the publisher, the editors and the reviewers. Any product that may be evaluated in this article, or claim that may be made by its manufacturer, is not guaranteed or endorsed by the publisher.
