# Supplementary material for: miR-190a-5p Partially Represses the Abnormal Electrical Activity of SCN3B in Cardiac Arrhythmias by Downregulation of IL-2
Source: Front Cardiovasc Med. 2022 Jan 10;8:795675. doi: 10.3389/fcvm.2021.795675 (PMC8784662; doi:10.3389/fcvm.2021.795675)
Supplement: Supplementary file 1 [file Data_Sheet_1.zip › Supplementary Figure 1.doc]

**Supplementary Figure 1.** Expression of *miR-190-5p* treated with *miR-190a-5p* mimic/inhibitor or miR-NC/inhibitor-NC in Raji cells.

(**A**) Expression of *miR-190a-5p* was upregulated by 422.22-fold when treated with *miR-190a-5p* (***P* < 0.01 *vs*. miR-NC); whereas it remained unchanged when transfected with *miR-190-5p* inhibitor (NS, no significant difference *vs*. inhibitor-NC). (**B**) mRNA expression of *IL-2* did not change when treated with *miR-190a-5p* mimic/inhibitor or miR-NC/inhibitor-NC (NS *vs*. miR-NC, NS *vs*. inhibitor-NC). (**C**) Concentration of IL-2 in culture medium of Raji cells decreased when treated with *miR-190a-5p* compared with miR-NC (***P* < 0.01 *vs*. miR-NC); however, concentration of IL-2 transfected with *miR-190a-5p* inhibitor was similar to that of inhibitor-NC (NS *vs*. inhibitor-NC). Data are shown as means ± SEM.
